# Myoclonic Epilepsy: Case Report of a Mild Phenotype in a Pediatric Patient Expanding Clinical Spectrum of *KCNA2* Pathogenic Variants

**DOI:** 10.3389/fneur.2021.806516

**Published:** 2022-02-01

**Authors:** Lorenzo Perilli, Gioia Mastromoro, Manuel Murciano, Ilaria Amedeo, Federica Avenoso, Antonio Pizzuti, Cristiana Alessia Guido, Alberto Spalice

**Affiliations:** ^1^Department of Mother and Child and Urological Sciences, Sapienza University of Rome, Rome, Italy; ^2^Faculty of Medicine and Dentistry, Department of Experimental Medicine, Sapienza University of Rome, Rome, Italy; ^3^Department of Emergency Pediatrics, Bambino Gesù Children's Hospital, IRCCS, Rome, Italy

**Keywords:** epilepsy, *KCNA2*, epileptic encephalopathies, genetic variants, genotype-first approach, epilepsy—abnormalities, classification, drug therapy

## Abstract

We report on the rare case of a male toddler presenting with myoclonic epilepsy characterized by daily episodes of upward movements of the eyebrows, and myoclonic jerks of both head and upper limbs. In addition, the child showed speech delay, tremors, and lack of motor coordination. Next Generation Sequencing analysis (NGS) performed in trio revealed in the proband the c.889C>T *de novo* missense variant in the *KCNA2* gene in heterozygous state. This is the first case of myoclonic epilepsy in a toddler due to a c.889C>T *KCNA2* missense variant. The patient was treated with valproic acid and ethosuximide with a good clinical response. At 6 years old, follow-up revealed that the proband was seizure-free with tremors and clumsiness in movements. According to the literature, this case supports the correlation between myoclonic epilepsy and *KCNA2* alterations. This evidence suggests that performing genomic testing including the *KCNA2* gene in preschool patients affected by myoclonic epilepsy, especially when associated with delayed neurodevelopment. Our goal is to expand the phenotypical spectrum of this rare condition and adding clinical features following a genotype-first approach.

## Introduction

Among the genetically determined forms of epilepsy, many genes remain unknown. Cases of epilepsy caused by a *KCNA2* mutation are known in literature (as shown in Table 1) different from the one reported in this manuscript. In 2017, Sachdev et al. (8) described a case of genetically determined epilepsy carrying the same mutation of our patient, but with different phenotype, described in Table 2. We report for the first time a pediatric patient affected by myoclonic epilepsy due to the heterozygous c.889C>T missense variant in the *KCNA2* gene. According to the literature, this case supports the correlation between myoclonic epilepsy and *KCNA2* alterations. This evidence suggests that performing genomic testing including the *KCNA2* gene in preschool patients affected by myoclonic epilepsy, especially when associated with delayed neurodevelopment. Our goal is to expand the phenotypical spectrum of this rare condition and adding clinical features following a genotype-first approach.

**Table 1:** Comparison between the characteristics of patients reported in the literature with mutation in the same triplet encoding an amino acid as the patient analyzed in this study.

	**Pena and Coimbra (1)**	**Syrbe et al. (2)**	**Corbett et al. (3)**	**Masnada et al. (4)**	**Masnada et al. (4)**	**Masnada et al. (4)**	**Masnada et al. (4)**	**Masnada et al. (4)**	**Canafoglia et al. (5)**	**Nashabat et al. (6)**	**Costain et al. (7)**
Case number	Case 1	Case 1	Case 1	Case 1	Case 2	Case 3	Case 4	Case 5	Case 1	Case 1	Case 1
Variant	c.890G>A, p.Arg297Gln *De novo*	c.890G>A, p.Arg297Gln *De novo*	c.890G>A, p.Arg297Gln *De novo*	c.890G>A, p.Arg297Gln *De novo*	c.890G>A, p.Arg297Gln (ovodonation)	c.890G>A, p.Arg297Gln *De novo*	c.890G>A, p.Arg297Gln *De novo*	c.890G>A, p.Arg297Gln (NR)	c.890G>A, p.Arg297Gln *De novo*	c.890 G > A, p.Arg297Gln (consanguinity)	c.890G>A, p.Arg297Gln *De novo*
Functional analisys	NR	Gain of function	Gain of function	NR	NR	NR	NR	NR	NR	NR	NR
Age of onset	15 m	5 m	12 m	10 m	15 m	since birth	6 m	12 m	36 m	20 m	8 m
Seizure type at onset	FS	Febrile SE	T, MC, AS	FS	FS	Infantile spasms	GTCS	GTCS	T	GTCS	T, GTCS, AS, MC
Other seizure types	MC, AS, GTCS	GTCS, AS	NR	GTCS, MC	MC, AS, GTCS	AS w/o MC, GTCS	AAS w/o MC	No	Prominent cortical myoclonus	No	NR
Clinical features	hypotonia and mild ataxia	Moderate-severe ataxia	psychomotor delay	Aggressiveness, stubbornness, psychomotor delay	Moderate ID, language delay, Stubbornness, difficulty of concentration, psychomotor delay	Moderate-severe ID and language delay, psychomotor delay (since birth)	Psychom. dev. delay (8–9 mo), ASD	Learning difficulties	Psychomotor delay, jerky movements of the upper limbs, clumsiness	N	N
Development at onset	Normal	Normal	N	N	Delayed	Delayed	N	Delayed	NR	N	N
EEG findings	BGS, irregular GSW; sleep activation	GSW and poly-Sp-W	GSW and polySp-W; bilateral posterior SW, BGS;	GSW, theta-beta activity + Sp in the midline	Slow background activity, Irregular GSW; sleep activation	BGS, GSW, posterior SW	BGS, right Occipital Sh-W, disorganized BG; irregular 2H GSW	BGS, GSW, Multifocal Epileptiform discharges	GSW; BGS, Sp on the posterior derivations, bilateral Sp during light sleep.	NR	Sp, SpW
Magnetic Resonance Imaging	NR	Normal	mild cerebellar atrophy	Severe cerebellar atrophy, small hippocampi	Severe cerebellar atrophy	N	Hyperintense subcortical white matter lesions	Cerebellar atrophy	Mild cerebellar atrophy, cisterna magna	Brain atrophy+cerebellar hypoplasia	Cerebellar atrophy
Neurological examination	ataxia and obvious delay of development	Moderate-severe ataxia, hyper-reflexia	Ataxia, cerebellar signs on examination, normal eye movement	Tremor, impaired coordination of fine motor skills, ataxia, dysarthria, myoclonia, pyramidal signs	N	Ataxia, finger tremor, impaired coordination	Tremor, ataxia, head titubation, axial hypotonia, pyramidal signs, impaired motor coordination	Impaired incoordination, mild dysdiadochokinesia, mild-moderate ataxia, dysarthria	Ataxia, irregularly repetitive myoclonic jerks during active hand movements	Ataxia	NR
Development at follow up	tremor of the extremities, loss of sphincter control and hyperkinetic behavior	Moderate ID	Slowing at 12 months	NR	NR	NR	NR	NR	Worsening of the movement disorder	Refractory to medications, still having seizure	NR

*NR, Not reported; GSW, generalized spike waves; Sp-W, spike-waves; Sp, spikes; Sh-W, sharp waves; BGS, background slowing; N, normal; ID, intellectual disability; ASD, autism spectrum disorder; S, febrile seizure; MC, Myoclonic convulsion; AS, absence seizures; T, tonic; AAS, atypical absence seizures; GTCS, generalized tonic seizures; SE, status epilepticus; EEG, electroencephalogram*.

**Table 2:** Comparison between the characteristics of the patient studied with the only previous case reported in the literature with the same mutation.

	**Sachdev et al. (8)**	**Our patient**
Variant	c.889 C>T, p.Arg297Trp *de novo*	c.889 C>T, p.Arg297Trp *de novo*
Functional analisys	Not reported	Not performed
Age of onset	4 years	4 years
Seizure type at onset	Generalized tonic seizures	Absence seizures
Other seizure types	Status epilepticus	Myoclonic convulsion
Development at onset	Normal	Speech delay
EEG findings	Slow posterior dominant rhythm activity, delta activity with Sp, Spike and Slow Waves in the bioccipital regions, Parasagittal ShW and Sp during activity, GSW, bifrontal ShW; GBS	Slow posterior dominant rhythm activity, theta activity with Sp on the parieto-occipital regions, GSW
MRI	Normal	Ectopy of the cerebellar tonsils (6 mm), hyperintensity of the deep white matter in the suvra/paratrigonal, in subcortical area and in the temporal area bilaterally
Neurological examination	Non-fluent language	Tremor of the hands, clumsiness in movement and in fine motricity
Development at follow up	Normal	Normal

*GSW, generalized spike waves; Sp, spikes; Sh-W, sharp waves; GBS, global background slowing; EEG, electroencephalogram*.

## Case Description

The proband was born at 38 weeks of gestational age through Cesarean section, performed due to maternal-fetal disproportion. Psychomotor development was characterized by autonomous walking at 16 months (referred balance disorder) and mild language delay improved at the age of 30 months after schooling. The patient has been fed with homogenized food up to 24 months of age and showed selectivity in choosing new ones. The parents reported frequent episodes of vomiting (once a week) without nausea, mostly after physical activity. At the age of 2, the boy experienced head trauma after loss of consciousness. Subsequently, the child showed generalized hypertonus, cyanosis, and deviation of the buccal rhyme that lasted about 2 min with self-resolution. The CT scan highlighted a skull fracture without encephalic lesions and a Chiari type 1 malformation, confirmed during the MRI that showed caudal ectopia of cerebellar tonsils (6 mm from Foramen Magnum), hyperintensity of the deep white matter in the suvra/paratrigonal, in subcortical area, and in the temporal area bilaterally, compatible with terminal areas of myelination. Based on the evidence of cerebellar tonsils ectopia, the toddler underwent clinic evaluation for hypermobility, but did not meet Beighton Criteria for Ehlers Danlos Syndrome diagnosis.

**Figure 1:**
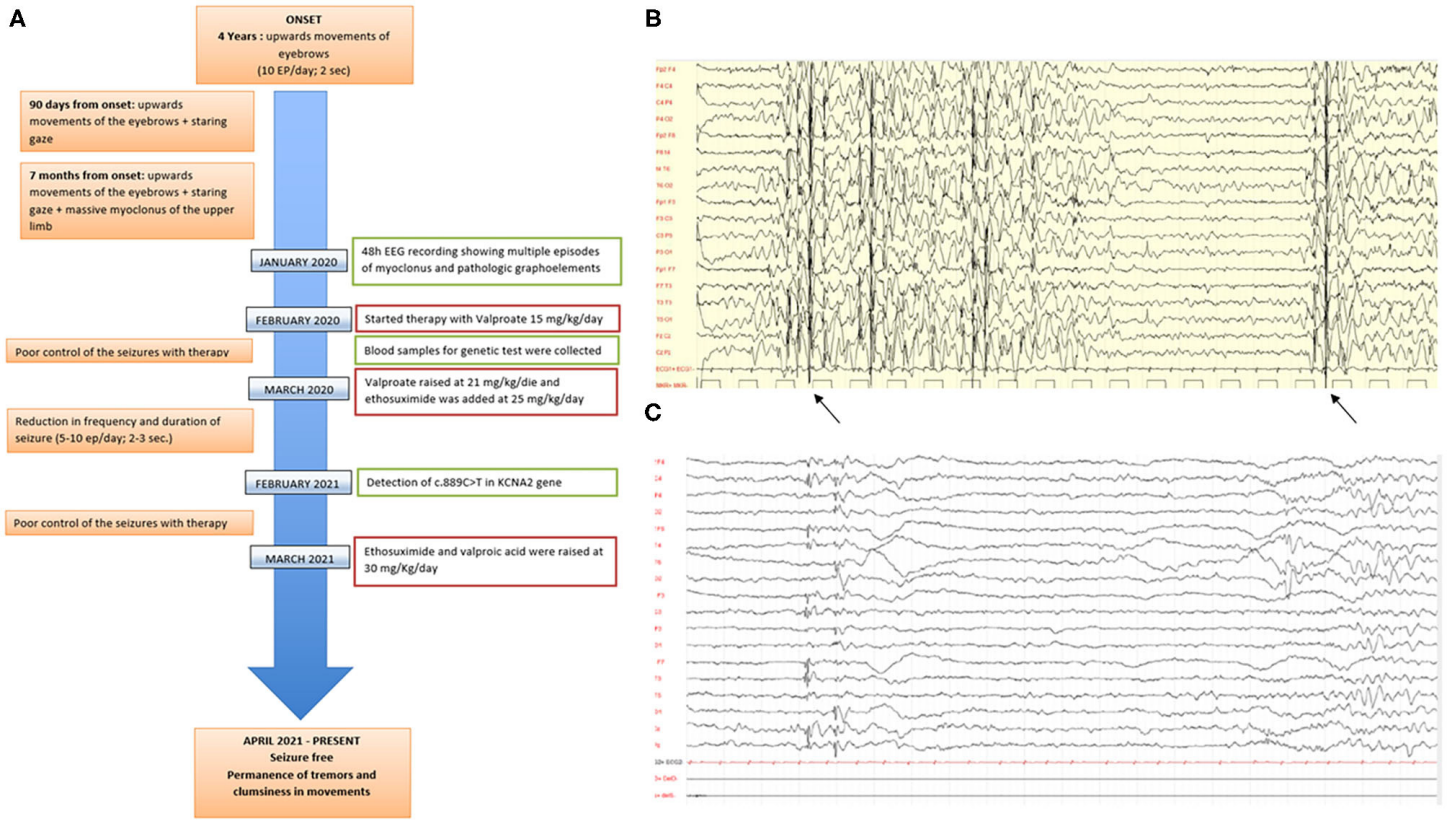
**(A)** Timeline of clinical features, instrumental characteristics, and therapy approaches in the proband since the toddler started showing epilepsy. **(B,C)** Electroencephalogram (EEG) performed when the child was 4 years old. The trace is characterized by poorly organized brain electrical activity, slow anomalies with interictal paroxysmal abnormalities on bilateral parieto-occipital sites, and abundant epileptiform anomalies with diffuse expression.

At the age of 4 years (Figure 1A), the child started experiencing daily episodes of upward movements of the eyebrows associated with staring spells. The first neurological examination showed tremor of both hands at the end of the index-nose test. The toddler asks for support while climbing stairs and when walking. Electroencephalogram (EEG) recordings while awake showed a trace characterized by poorly organized brain electrical activity and differentiated by age, slow anomalies with interictal paroxysmal abnormalities on bilateral parieto-occipital regions, and abundant epileptiform anomalies with diffuse expression, the most prolonged associated with clinical correlates (Figures 1B,C). The physical examination does not underline any dysmorphism except for the presence of flaring of the lateral side of the eyebrows. After 3 months, frequency and duration of the seizures had increased (up to 20 episodes/day in clusters), subsequently associated with massive ictal myoclonus of the upper limbs or head of very short duration (80 episodes/day in clusters). A 48-h EEG recording showed multiple episodes of myoclonus and pathologic graphoelements.

Next Generation Sequencing analysis (NGS) performed in trio on a panel of 77 genes related to epilepsy revealed the missense variant c.889C>T; p.Arg297Trp: NM_004974 in the third exon of the KCNA2 gene (potassium channel, voltage-gated, shaker-related subfamily, member 2, chr1:110,593,579-110,631,535;GRCh38, MIM ^*^176262) in a heterozygous state. Sanger sequencing did not detect this variant in parental DNA, suggesting a *de novo* origin of the alteration.

So, the toddler started antiepileptic therapy with sodium valproate at a dosage of 15 mg/kg/day which required, given the poor control of events, a raise to 21 mg/kg/day. This therapy did not result in any change of the clinical picture and EEG pattern. Therefore, after about 2 months of treatment, ethosuximide was added at a dosage of 25 mg/day with a reduction in the seizures and EEG abnormalities. Approximately a year later, due to the poor control of the therapy, valproic acid was raised to 30 mg/Kg/day and ethosuximide to 30 mg/Kg/day with complete resolution of the pathological picture.

At the age of 5 years and 11 months, the toddler has been tested with WPPSI-III, CPM, CBCL, and ABAS II. Based on the evaluation carried out, a normal cognitive functioning emerges (IQ 91), with abilities adequate to the expected level in the verbal performance and general language scale.

There is a significant decline in the domain that evaluates the processing speed. Non-verbal intelligence is adequate for the chronological age. Assessment of the adaptive profile, as reported by the mother of child, showed subnormal scores in the conceptual, social, and practical domains. Critical issues were observed in the categories related to preschool skills, communication, self-control, play, and use of the environment. Currently, the proband presents no epileptic events and improvement in the EEG picture, which still maintains theta-delta slow wave bursts in the occipital regions. Neurological examination appears to be normal, except for tremor of the hands and mild fine motor skills deficit. Some behavioral problems persist. In the EEG assessments carried out from 2017 to 2021, it was evident that the permanence of the slow theta-delta variable rhythm anomalies in the posterior regions, with isolated spike-and-wave complexes on the parieto-occipital sites bilaterally, sometimes fronto- or temporo-central that tend to present in diffuse paroxysms with diffuse expression of varying duration. The associated clinical correlation consists in a reduction in contact, ideomotor slowdown with spontaneous resolution, and asynchronous four-limb myoclonus. Overall, the EEG picture appeared disorganized for the age of child. After 7 months being seizure free, in the latest evaluation, the child no longer presents generalized epileptiform abnormalities.

The psychological tests carried out showed performance at the normal range in the ability to analyze and synthesize visual stimuli based on correct visual perception and visuomotor coordination. In addition, they highlighted results in the average range in logical reasoning and in the ability to abstract reason and categorize visual stimuli. The child showed good expressive and receptive language skills. The scale that assesses skills related to comprehension of verbal terms and instructions associated with long-term memory reached levels above normal. There was a decline in the ability to perform cognitive tasks smoothly and automatically especially under the pressure to maintain focused attention and concentration. Scores showed significantly below normal abilities related to the visual discrimination associated with visual short-term memory and visual-motor coordination and cognitive flexibility; even the ability to work quickly with unusual material achieved below normal performance. The toddler presented a cognitive profile that is adequate to the expected level for gender and age, although there is a significant drop in the domain that assesses the speed of processing. The adaptive framework presents criticalities that currently do not seem to affect the life of the child.

## Discussion

This is the first case of a toddler affected by myoclonic epilepsy due to a *de novo* missense pathogenic variant (c.889C>T) in the *KCNA2* gene in the heterozygous state.

The *KCNA2* gene encodes for 2 NCBI Refseq transcripts (NM_004974.4 and NM_001204269.2). Monoallelic *KCNA2* pathogenic variants, encoding the voltage-gated K+ channel Kv1.2, have been reported as a cause of developmental delay and seizures (Developmental and epileptic encephalopathy 32, MIM #616366) (1–3, 9–13).

Mice models, carrying a *KCNA2* mutation, show motor incoordination, myoclonic jerks, tremor, and small body size (14), while null animals present increased seizure susceptibility (15). Previous functional studies showed that *KCNA2* mutations cause either a dominant-negative loss-of-function, or a drastic gain-of function (2), determining distinct phenotypes in patients (4, 16). Alterations, causing loss-of-function, are characterized by a better prognosis than gain-of-function and mixed forms. In dominant-negative loss-of-function, focal predominant seizures with greater sleep activation are reported in literature. In gain-of-function, critical events seem to be prevalently severe generalized seizures, most frequently are problems in neurodevelopment, such as ataxia. At a structural level, the cerebellar or whole brain atrophy has been frequently reported. Severe early onset epilepsies appear to be more frequent in mixed forms. Neonatal onset epilepsies with developmental impairments or generalized ictal events are rare (4, 16, 17). Recently the phenotypic spectrum has been expanded to include forms of progressive myoclonus epilepsy and myoclonic-atonic epilepsy (18) and functional studies of pathogenic variants had been performed (19).

The p.Arg297Trp variant, detected in the present case, is ranked as “Likely Pathogenic” according to the American College of Medical Genetics guidelines and “Pathogenic” according to ClinVar. Allele frequency is not available. The case we describe here represents the first in pediatric age and the second overall case of myoclonic epilepsy caused by this heterozygous pathogenic variant in *KCNA2*.

The toddler presented at the onset generalized epileptiform abnormalities that were often associated with upward movements of the eyebrows, fixation gaze, and ictal myoclonus of head and upper limbs. The toddler still presents difficulties in autonomous walking and while climbing stairs. The parents reported weekly vomiting episodes not preceded by nausea mostly associated with physical exercise, that could represent a clinical manifestation of seizure. After the therapeutical adjustments, given the considerable reduction in ictal events and the inability to perform a functional analysis of the gene, it has not been possible to attempt therapeutic strategies as recently suggested by the literature, such as 4-aminopyridine (20).

During sleep EEG showed fast/slow wave and polypoint/slow wave of medium- or large-voltage, on the fronto-central regions bilaterally, electrical status epilepticus during sleep (ESES) was not present as described by previous authors (21).

A literature review was conducted to identify the characteristics of reported patients who presented a mutation at the same triplet encoding an amino acid (as shown in Table 1). A case with the same mutation was described (8): the patient, differently from our toddler, was diagnosed with unprovoked generalized tonic-clonic seizures. Age of onset was 4 years old in a normal psychomotor neurodevelopment. After carbamazepine treatment, the boy showed seizure remittance. The toddler was worsened from 19 years old, when started presenting episodes of refractory status epilepticus strongly drug resistant. Similarly to our patient, EEG at the age of 4 was characterized by continuous polymorphic delta and theta slowing of the posterior dominant rhythm, intermittent bursts of rhythmic 2–2.5 Hz delta activity intermixed with spikes, spike-and-slow wave discharges over the bi-occipital regions, and isolated parasagittal sharp wave and spike activity during sleep.

Sachdev et al. (8) compared the similar clinical picture of their patient carrying the c.889 C>T mutation, to the reported cases detected with the c.890 G>A mutation in the same amino acidic site, differently from our patient that presents a mild clinical history. In Table 2, we compare the characteristics of the patient analyzed in this study with the only previous case reported in the literature with the same mutation (8).

Several cases detected with the c.890G>A p.(Arg297Gln) variant in the KCNA2 gene had been reported, expanding the clinical spectrum of developmental and epileptic encephalopathy and highlighting the variable expressivity. Progressive myoclonic epilepsy was described in one of the patients described by Canafoglia (5). This case showed ataxia and psychomotor delay, whereas epilepsy was delayed and limited to relatively rare tonic seizures, associated with prominent cortical myoclonus, occurred at 3 years. Syrbe et al. (2) reported the case of a toddler carrying a c.890G>A p.(Arg297Gln) *de novo* mutation in the *KCNA2* gene, whose functional analysis showed a gain-of-function alteration. The patient, differently from the reported case, experienced generalized tonic-clonic seizures, absences, status epilepticus often during febrile events. Moreover, the patient presented moderate-severe ataxia and moderate intellectual disability. The same nucleotidic variant was described in a patient with myoclonic and absence seizures. The age of onset, the clinical, and the neuropsychological deterioration was different from our case, resulting in a progressive worsening and drug resistance.

Masnada et al. (4) enrolled five patients carrying this mutation in the KCNA2 gene. Differently from the present case, all of them showed generalized tonic-clonic seizures, but they all presented tremors, ataxia, and a moderate intellectual disability. Two of them experienced myoclonic seizures associated with absences. Only one was seizure-free from 18 months of age. Corbett et al. (3) reported a patient that started having tonic seizures at 12 months, progressing in erratic myoclonic tremors. The proband showed different ictal phenotypes, while EEG and the neuropsychological evaluation showed similar results. Nashabat et al. (6) described the case of a patient that, differently from the present case, experienced earlier onset of generalized tonic-clonic seizures in a good global development, but with poor response to therapy. Costain et al. (7) reported a patient showing various ictal events, such as generalized tonic and tonic-clonic seizures, absences, and myoclonic seizures. This toddler presented an earlier age of onset, a good global development, and ataxia. Ngo et al. (22) described a case with apparently isolated ataxia as primary symptom, but no more clinical information was provided.

Interestingly, an unusual case of mosaicism of two novel missense variants in *KCNA2* had been reported, expanding the phenotypic spectrum associated with this mutations of gene, but not presenting with myoclonic epilepsy (23).

## Conclusions

The present case aims to expand the clinical spectrum associated with heterozygous *KCNA2* pathogenic variants. This evidence suggests a genotype-first approach, performing genomic tests that include the *KCNA2* gene, in patients with myoclonic epilepsy diagnosis. According to the literature, we highlight the variability in expression that can be clinically observed in patients with heterogeneous ictal events (15), even in those who show mutations in the same triplet encoding an amino acid. Indeed, the patient we describe presented a good prognosis characterized by a good response to therapy, and an improved neurodevelopment, in contrast with the previously reported cases, presenting with a more severe clinical picture (1, 2, 11).

We suggest that an accurate clinical examination improve the detection of genotype-phenotype correlations and expand the possibility of predicting the functional impact of *KCNA2* variants (18). Even if it is a rare condition, a *KCNA2* gene mutation test should be performed in patients over 3 years of age.

## Data Availability Statement

The raw data supporting the conclusions of this article will be made available by the authors, without undue reservation.

## Ethics Statement

Ethical review and approval was not required for the study on human participants in accordance with the local legislation and institutional requirements. Written informed consent to participate in this study was provided by the participants' legal guardian/next of kin.

## Author Contributions

LP, GM, MM, IA, FA, AP, CG, and AS: conceptualization, data curation, resources, investigation, writing—original draft, methodology, visualization, supervision, and writing—review and editing. All authors contributed to the article and approved the submitted version.

## Funding

All phases of this study were supported by the Department of Pediatrics of Sapienza University of Rome, Italy.

## Conflict of Interest

The authors declare that the research was conducted in the absence of any commercial or financial relationships that could be construed as a potential conflict of interest.

## Publisher's Note

All claims expressed in this article are solely those of the authors and do not necessarily represent those of their affiliated organizations, or those of the publisher, the editors and the reviewers. Any product that may be evaluated in this article, or claim that may be made by its manufacturer, is not guaranteed or endorsed by the publisher.

## References

[1] Pena SD Coimbra RL. Ataxia and myoclonic epilepsy due to a heterozygous new mutation in KCNA2: proposal for a new channelopathy. Clin Genet. (2015) 87:e1–3. 10.1111/cge.12542 25477152

[2] Syrbe S Hedrich UBS Riesch E Djémié T Müller S Møller RS. *De novo* loss- or gain-of function mutations in KCNA2 cause epileptic encephalopathy. Nat Genet. (2015) 47:393–9. 10.1038/ng.3239 25751627 PMC4380508

[3] Corbett MA Bellows ST Li M Carroll R Micallef S Carvill GL. Dominant KCNA2 mutation causes episodic ataxia and pharmacoresponsive epilepsy. Neurology. (2016) 87:1975–84. 10.1212/WNL.0000000000003309 27733563 PMC5109949

[4] Masnada S Hedrich UBS Gardella E Schubert J Kaiwar C Klee EW. Clinical spectrum and genotype-phenotype associations of KCNA2-related encephalopathies. Brain. (2017) 140:2337–54. 10.1093/brain/awx184 29050392

[5] Canafoglia L Castellotti B Ragona F Freri E Granata T Chiapparini L. Progressive myoclonus epilepsy caused by a gain-of-function KCNA2 mutation. Seizure. (2019) 65:106–8. 10.1016/j.seizure.2019.01.005 30660924

[6] Nashabat M Al Qahtani XS Almakdob S Altwaijri W Ba-Armah DM Hundallah K. The landscape of early infantile epileptic encephalopathy in a consanguineous population. Seizure. (2019) 69:154–72. 10.1016/j.seizure.2019.04.018 31054490

[7] Costain G Cordeiro D Matviychuk D Mercimek-Andrews S. Clinical application of targeted next-generation sequencing panels and whole exome sequencing in childhood epilepsy. Neuroscience. (2019) 418:291–310. 10.1016/j.neuroscience.2019.08.016 31487502

[8] Sachdev M Gaínza-Lein M Tchapyjnikov D Jiang YH Loddenkemper T Mikati MA. Novel clinical manifestations in patients with KCNA2 mutations. Seizure. (2017) 51:74–6. 10.1016/j.seizure.2017.07.018 28806589

[9] Tang S Addis L Smith A Topp SD Pendziwiat M Mei D. Phenotypic and genetic spectrum of epilepsy with myoclonic atonic seizures. Epilepsia. (2020) 61:995–1007. 10.1111/epi.16508 32469098

[10] Allen NM Conroy J Shahwan A Lynch B Correa RG Pena SD. Unexplained early onset epileptic encephalopathy: exome screening and phenotype expansion. Epilepsia. (2016) 57:e12–7. 10.1111/epi.13250 26648591

[11] Hundallah K Alenizi A AlHashem A Tabarki B. Severe early-onset epileptic encephalopathy due to mutations in the KCNA2 gene: expansion of the genotypic and phenotypic spectrum. Eur J Paediatr Neurol. (2016) 20:657–60. 10.1016/j.ejpn.2016.03.011 27117551

[12] Drögemöller BI. Maintaining the balance: both gain- and loss-of-function KCNA2 mutants cause epileptic encephalopathy. Clin Genet. (2015) 88:137–9. 10.1111/cge.12615 25997620

[13] Allou L Julia S Amsallem D El Chehadeh S Lambert L Thevenon J. Rett-like phenotypes: expanding the genetic heterogeneity to the KCNA2 gene and first familial case of CDKL5-related disease. Clin Genet. (2017) 91:431–40. 10.1111/cge.12784 27062609

[14] Xie G Harrison J Clapcote SJ Huang Y Zhang JY Wang LY. A new Kv1.2 channelopathy underlying cerebellar ataxia. J Biol Chem. (2010) 285:32160–73. 10.1074/jbc.M110.153676 20696761 PMC2952217

[15] Brew HM Gittelman JX Silverstein RS Hanks TD Demas VP Robinson LC. Seizures and reduced life span in mice lacking the potassium channel subunit Kv1.2, but hypoexcitability and enlarged Kv1 currents in auditory neurons. J Neurophysiol. (2007) 98:1501–25. 10.1152/jn.00640.2006 17634333

[16] Morrison-Levy N Borlot F Jain P Whitney R. Early-onset developmental and epileptic encephalopathies of infancy: an overview of the genetic basis and clinical features. Pediatr Neurol. (2021) 116:85–94. 10.1016/j.pediatrneurol.2020.12.001 33515866

[17] Steel D Symonds JD Zuberi SM Brunklaus A. Dravet syndrome and its mimics: beyond SCN1A. Epilepsia. (2017) 58:1807–16. 10.1111/epi.13889 28880996

[18] Döring JH Schröter J Jüngling J Biskup S Klotz KA Bast T. Refining genotypes and phenotypes in KCNA2-related neurological disorders. Int J Mol Sci. (2021) 22:2824. 10.3390/ijms22062824ijms22062824 33802230 PMC7999221

[19] Arbini A Gilmore J King MD Gorman KM Krawczyk J McInerney V. Generation of three induced pluripotent stem cell (iPSC) lines from a patient with developmental epileptic encephalopathy due to the pathogenic KCNA2 variant c.869T>G; p.Leu290Arg (NUIGi052-A, NUIGi052-B, NUIGi052-C). Stem Cell Res. (2020) 46:101853. 10.1016/j.scr.2020.101853 32540721

[20] Imbrici P Conte E Blunck R Stregapede F Liantonio A Tosi M. A novel *KCNA2* variant in a patient with non-progressive congenital ataxia and epilepsy: functional characterization and sensitivity to 4-aminopyridine. Int J Mol Sci. (2021) 22:9913. 10.3390/ijms22189913 34576077 PMC8469797

[21] Gong P Xue J Jiao X Zhang Y Yang Z. Genetic etiologies in developmental and/or epileptic encephalopathy with electrical status epilepticus during sleep: cohort study. Front Genet. (2021) 12:607965. 10.3389/fgene.2021.607965 33897753 PMC8060571

[22] Ngo KJ Rexach JE Lee H Petty LE Perlman S Valera JM. A diagnostic ceiling for exome sequencing in cerebellar ataxia and related neurological disorders. Hum Mutat. (2020) 41:487–501. 10.1002/humu.23946 31692161 PMC7182470

[23] Gong P Jiao X Zhang Y Yang Z. Complex mosaicism of two distinct mutations in a female patient with *KCNA2*-related encephalopathy: a case report. Front Genet. (2020) 11:911. 10.3389/fgene.2020.00911 32903602 PMC7438874

